# The Effect of Inter-pulse Interval on TMS Motor Evoked Potentials in Active Muscles

**DOI:** 10.3389/fnhum.2022.845476

**Published:** 2022-03-22

**Authors:** Noora Matilainen, Marco Soldati, Ilkka Laakso

**Affiliations:** ^1^Department of Electrical Engineering and Automation, Aalto University, Espoo, Finland; ^2^Aalto Neuroimaging, Aalto University, Espoo, Finland

**Keywords:** TMS, inter-pulse interval, motor evoked potential, motor mapping, active muscle contraction, motor threshold

## Abstract

**Objective:**

The time interval between transcranial magnetic stimulation (TMS) pulses affects evoked muscle responses when the targeted muscle is resting. This necessitates using sufficiently long inter-pulse intervals (IPIs). However, there is some evidence that the IPI has no effect on the responses evoked in active muscles. Thus, we tested whether voluntary contraction could remove the effect of the IPI on TMS motor evoked potentials (MEPs).

**Methods:**

In our study, we delivered sets of 30 TMS pulses with three different IPIs (2, 5, and 10 s) to the left primary motor cortex. These measurements were performed with the resting and active right hand first dorsal interosseous muscle in healthy participants (*N* = 9 and *N* = 10). MEP amplitudes were recorded through electromyography.

**Results:**

We found that the IPI had no significant effect on the MEP amplitudes in the active muscle (*p* = 0.36), whereas in the resting muscle, the IPI significantly affected the MEP amplitudes (p < 0.001), decreasing the MEP amplitude of the 2 s IPI.

**Conclusions:**

These results show that active muscle contraction removes the effect of the IPI on the MEP amplitude. Therefore, using active muscles in TMS motor mapping enables faster delivery of TMS pulses, reducing measurement time in novel TMS motor mapping studies.

## 1. Introduction

Transcranial magnetic stimulation (TMS) is a useful tool for motor mapping. Cortical motor maps help identifying lesions or plasticity changes in the motor system (Lefaucheur, 2019), and they are also used for presurgical assessment of brain tumor surgery (Lefaucheur and Picht, 2016).

Recently developed methods aim to localize the effect of TMS in the cerebral cortex using computer simulations of the induced electric fields (Bungert et al., 2017; Laakso et al., 2018; Weise et al., 2020). These methods, however, require a large number of pulses lengthening the measurement time. A common practice is to use fairly long inter-pulse intervals (IPIs) in order to avoid the effect of the IPI on motor evoked potential (MEP) amplitudes (Julkunen et al., 2012; Vaseghi et al., 2015; Pellicciari et al., 2016; Hassanzahraee et al., 2019). However, the effect is reported only for resting muscles. For example, Bungert et al. (2017), Laakso et al. (2018) and Kataja et al. (2021) used active muscle contraction in their studies. Furthermore, there is some indication that the IPI has no effect on responses evoked in active muscles (Möller et al., 2009). Möller et al. discovered that the hysteresis effect, which was observed with a resting muscle, did not occur when the muscle was active. In addition, previous studies have suggested more thorough investigation of the effect on MEP amplitude when using an active muscle, as it has not been studied before (Vaseghi et al., 2015; Hassanzahraee et al., 2019).

Future research would benefit from the use of a shorter IPI for active muscles. Therefore, the objective of this study is to investigate the possibility of using active muscle contraction in TMS motor mapping with a shorter IPI in order to reduce the measurement time.

## 2. Materials and Methods

### 2.1. Participants

The data was collected from 13 healthy participants who were right handed by self report and participated in two experimental conditions, active and resting. One participant was excluded from the study because of a high motor threshold. Two participants were excluded from the active condition as the baseline muscle activity was not sufficient. The resting condition was not performed for three participants. Altogether, seven participants were included in both the active and resting condition. Finally, the analysis of the active condition data included 10 participants (7 male, 3 female, mean age ± SD = 30.8 ± 5.8, age range: 25–40) and the analysis of the resting condition data included 9 participants (7 male, 2 female, mean age ± SD = 30.1 ± 5.6, age range: 22–40). All participants gave their written consent for participation. The study was approved by the Aalto University Research Ethics Committee.

### 2.2. Magnetic Resonance Imaging

T1 and T2 weighted magnetic resonance (MR) images were acquired using a 3 T MRI scanner (Magnetom Skyra; Siemens, Ltd., Erlangen, Germany). The imaging parameters are listed as follows. T1: TR/TE/TI/FA/FOV/voxel size/slice number = 1,800/1.99/800 ms/9°/256/1 × 1 × 1 mm/176; and T2: TR/TE/FOV/voxel size/slice number = 3,200/412 ms/256/ 1 × 1 × 1 mm /176. The data have been measured at AMI Centre, Aalto NeuroImaging, Aalto University School of Science.

### 2.3. Cortical Reconstruction and TMS Coil Location

The coil locations for the experiments were determined in advance to target the stimulation to the first dorsal interosseus (FDI) target location in the hand area of the left hemisphere.

First, cortical reconstructions were generated from the T1-weighted MR images using the FreeSurfer image analysis software (Dale et al., 1999; Fischl et al., 1999). After reconstruction, FreeSurfer was used to generate a mapping between the reconstructed surface of the individual brain and the surface reconstruction of the Montreal Neurological Institute (MNI) ICBM 2009a nonlinear asymmetric template brain (Fonov et al., 2009, 2011).

**Figure 1:**
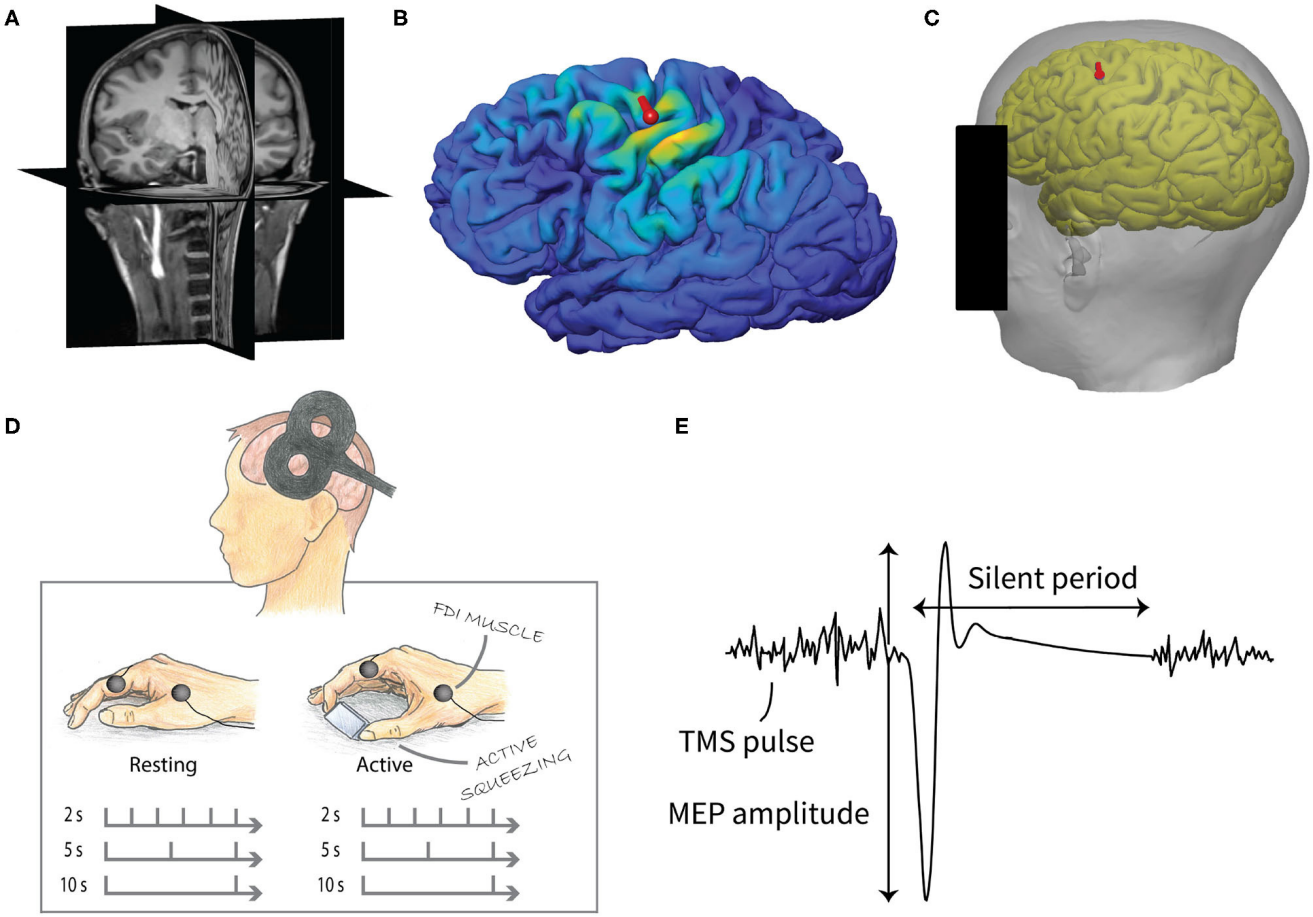
Experimental design of the study. **(A)** T1- (pictured) and T2-weighted MR images were used to create individual cortical reconstructions. **(B)** The induced electric fields were calculated based on computer simulations. **(C)** The location of the TMS coil (red mark) for the experiment was predetermined from the cortical reconstruction in order to obtain the optimal cortical location for the FDI muscle. **(D)** TMS measurement consisted of two parts, resting and active condition. Both included three sets of 30 stimuli with 2, 5, and 10 s IPI. The order of the conditions and IPIs were pseudo-randomized. **(E)** MEPs were recorded from the FDI muscle of the right hand. The peak-to-peak amplitude of the MEPs was used in the analysis. The silent period was defined as the duration between the MEP onset and the resumption of the voluntary EMG.

For each participant, the mapping was used to obtain an individual cortical target location that corresponded to [−41, −7, 63] in MNI coordinates, which was previously estimated to be the group-average activation site for the FDI muscle (Laakso et al., 2018). The coil was positioned on the scalp at the closest point to the selected target cortical location. The coil orientation was selected so that the induced current direction was approximately perpendicular to the course of the central sulcus in the posterior-anterior direction at the target location. Finally, the predetermined coil locations and directions were marked on the MR images, which were used for neuronavigation (Figures 1B,C).

### 2.4. TMS and EMG Recordings

TMS was performed with a monophasic Magstim 200^2^ stimulator (Magstim Company, UK) using an eight-shaped coil, which consists of two adjacent round wings of 9 cm diameter. The location and orientation of the coil were tracked and recorded with the Visor2 TMS neuronavigation system (ANT Neuro, Enschede, the Netherlands). The data have been measured at Aalto TMS, Aalto NeuroImaging, Aalto University School of Science.

Resting and active motor threshold (RMT and AMT) intensities were defined as the lowest intensities required to elicit TMS MEPs (peak-to-peak amplitude of >50 μ*V* with resting and 100 μ*V* with active condition) in at least 50% of successive trials (Rossini et al., 2015). MEPs were recorded from the right hand FDI muscle with the NeurOne EMG system (NeurOne, MEGA Electronics Ltd, Finland) and disposable Ag/AgCl surface electrodes. The EMG signal was sampled at 5 kHz and high-pass filtered with a 10 Hz cutoff frequency.

### 2.5. Experimental Setup

We studied the MEPs measured from the right hand FDI muscle when the primary motor cortex was stimulated by TMS. Three different IPIs (2, 5, and 10 s) with two different conditions, active and resting muscle, were used. Thirty pulses were delivered for each IPI for both conditions using a stimulation intensity approximately 20% above the motor threshold intensity. The order of the IPIs and conditions were pseudo-randomized. The experimental setup is illustrated in Figure 1.

During the measurement, the participant was sitting on a chair with the magnetic coil positioned using a mechanical holder on the predetermined scalp location above their left cerebral hemisphere. Their right arm was resting on a pillow placed on their lap. In the resting condition, the participant kept their hand resting on a pillow. In the active condition, their task was to contract their FDI muscle by applying a constant pressure on a small object with their index finger and thumb while their hand was still. Participants were instructed to observe their EMG activation from the screen in front of them and keep the peak-to-peak amplitude close to 200 μV.

### 2.6. Calculation of the Induced Electric Field

The finite-element method was used to computationally estimate the induced electric field at the cortical target location. The details of the computer simulations were similar to our previous study (Laakso et al., 2018). Briefly, volume conductor models were generated from the cortical reconstructions generated using FreeSurfer and the segmentation of the T1- and T2-weighted MR images. The following electric conductivity values were assigned to the segmented tissues and bodily fluids (unit: S/m): gray matter (0.215), white matter (0.142), cerebrospinal fluid (1.79), compact and spongy bone (0.009 and 0.034), subcutaneous fat (0.15), scalp (0.43), muscle (0.18), dura mater (0.18), and blood (0.7).

A model of the figure-8 coil (Çan et al., 2018) was placed on the location recorded in the experiments using the neuronavigation system. We note that the recorded location might differ slightly from the predetermined scalp location. The induced electric field was determined using the FEM with a uniform grid of first-order cubical elements with a side length of 0.5 mm (Laakso and Hirata, 2012). Finally, the electric field magnitude was calculated at the individual target cortical location. In addition, we calculated the maximum value of the electric field magnitude in the cortical gray matter at a depth of 2 mm below the pial surface.

### 2.7. Data Processing and Statistical Analysis

The MEP amplitude was defined as the peak-to-peak distance between the negative and positive peak in the waveform. The EMG baseline value for active condition was defined as the root mean square of the EMG signal in one second interval before the stimulus. The length of the silent period was defined as the duration between the MEP onset and the resumption of the voluntary EMG.

A linear mixed-effects model was used to predict the relationship between the MEP amplitude and the IPI. This model allows non-independent observations and considers inter-subject variability as well as the EMG baseline level. For the analysis, the MEP amplitude was log transformed in order to ensure the normality of the residuals. Analyses were performed with the open-source programming language R (R Core Team, 2013), separately for the resting and active conditions.

For the resting condition, the model included the IPI (2, 5, and 10 s), pulse number (1–30) and their interaction as fixed effects. Participants were treated as a random effect. The order of the IPI measurement appeared non-significant when included in the model and did not result in a better model fit using the Akaike information criterion. Therefore, it was excluded from the model making the final model simpler. For the active condition, an additional fixed effect, EMG baseline, was included in the model, as the active muscle contraction causes a slightly varying baseline level that can affect the MEP. Maximum likelihood was used as the estimation method for the model coefficients. *P*-values were obtained by likelihood ratio tests of the full models with the effect of the IPI against the null model (without the IPI).

*Post-hoc* analyses were conducted to compare the different IPIs by the means of a Wilcoxon signed-rank test (as the data is dependent) with a Bonferroni adjustment of the p-values. A p-value smaller than 0.05 was considered significant for all statistical tests. For visualization and *post-hoc* analyses, the participant specific intercepts were removed from the data using the linear mixed-effects model.

To study whether the IPI affected the variability of the MEP amplitudes between trials, the coefficient of variation (CV = 100 × SD/mean) of the log transformed MEP amplitudes was computed for each participant at each IPI and condition (active and resting).

Additionally, a linear mixed-effects model was used to predict the relationship between the length of the silent period and the IPI. For the analysis, the length was log transformed in order to ensure the normality of the residuals.

## 3. Results

### 3.1. Motor Threshold and Induced Electric Field

Intensities for the RMT and AMT were 43.2±5.2% and 38.8±7.2% (mean ± SD) of the maximum stimulator output, respectively. The corresponding induced electric field magnitudes in the gray matter at the predetermined FDI target location were 135±40 and 108±37 V/m (mean ± SD) for the resting and active conditions, respectively.

The corresponding maximum values of the electric field magnitude in the gray matter were 188±46 and 151±37 V/m (mean ± SD). The maximum values did not differ significantly from those reported in an earlier study (Laakso et al., 2018) (Student's *t*-tests, p = 0.2 and p = 0.07, respectively), where the magnetic coil was manually positioned by searching the “hotspot” for the FDI muscle. This indicated that the coil location used in the experiments was comparable to that obtained using the conventional method.

### 3.2. Effects of Inter-pulse Interval on MEP Amplitude

**Figure 2:**
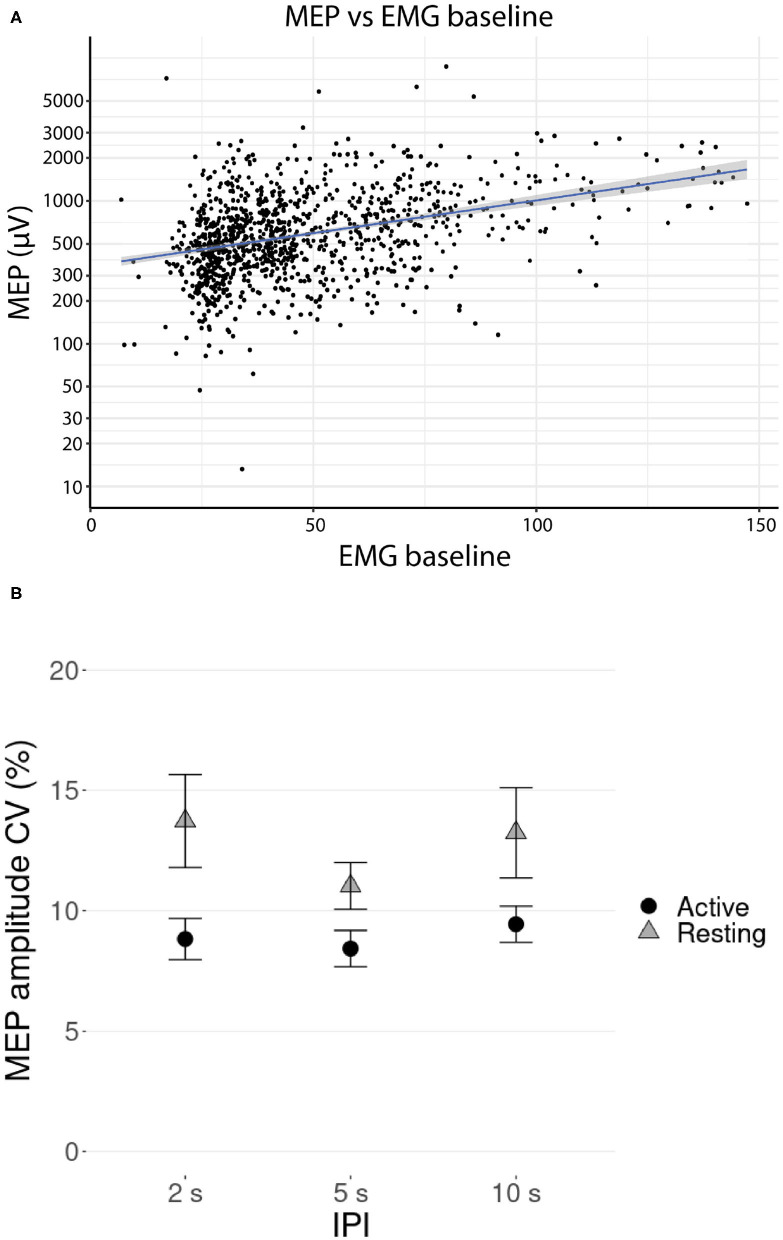
**(A)** Relationship between the MEP amplitudes and the EMG baseline (RMS value over 1 s before the stimulus) during TMS stimulation with active contraction on logarithmic scale. Participant-specific intercepts have been removed from the data using a linear mixed effects model. The gray shaded area around the regression line represents the 95% confidence interval. **(B)** Mean coefficient of variation (CV) of the log transformed MEP amplitude versus IPI at active and resting conditions. Error bars represent the standard errors.

A linear mixed-effects model with a likelihood ratio test showed that the IPI had a significant effect on the MEP amplitude for the resting [χ^2^(2) = 18.91, p < 0.001] but not for the active muscle [χ^2^(2) = 2.02, p = 0.36]. The only fixed effect significantly affecting the MEP amplitudes during active muscle contraction was the baseline EMG magnitude before the pulse [χ^2^(1) = 85.20, p < 0.001], a higher baseline producing higher amplitudes. The relationship between the MEP amplitude and the EMG baseline is visualized in Figure 2A.

Mean coefficient of variation (CV) of the log transformed MEP amplitude is presented in Figure 2B. *Post-hoc* testing showed that the CVs were significantly lower at active condition than at resting condition (pairwise Wilcoxon signed-rank tests, p < 0.01, Bonferroni corrected), but there was no support for significant differences in CVs between the IPI groups (pairwise Wilcoxon signed-rank tests, all p >0.32, Bonferroni corrected).

**Figure 3:**
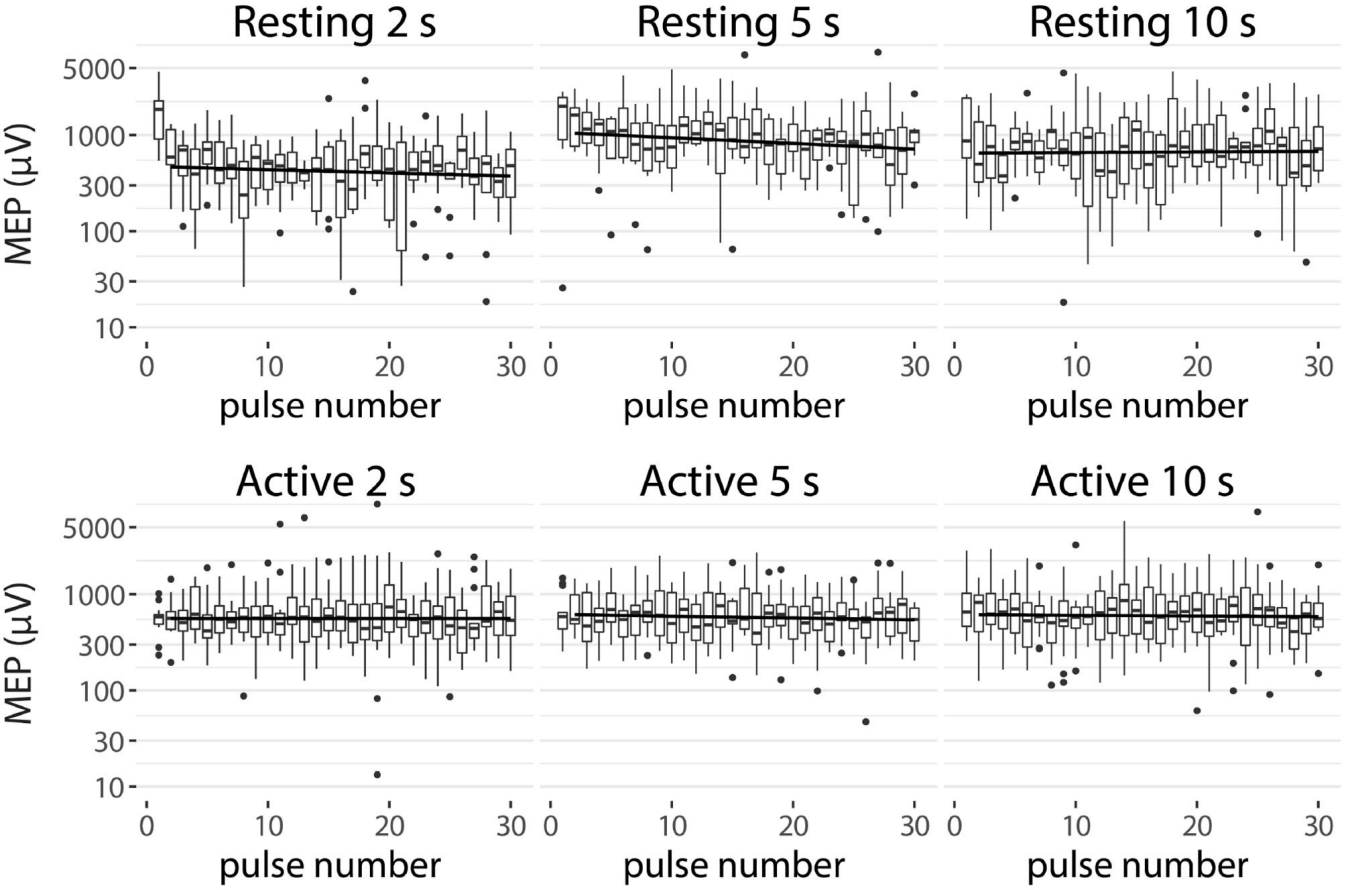
MEP amplitudes of each IPI (2, 5, and 10 s) for resting and active condition. Participant-specific intercepts have been removed from the data using a linear mixed effects model. Graphs represent the minimum, maximum, median, first quartile, and third quartile in the data set on a logarithmic scale. The trend line is drawn from the second to the last pulse.

The effect of the pulse number on the MEP amplitude for each IPI is illustrated in Figure 3. For the resting condition, the MEP amplitude of the first pulses appear to be higher than the MEP amplitude of the later pulses in the 2 and 5 s IPIs. For the 2 s IPI, *post-hoc* analysis shows a significant difference (pairwise Wilcoxon signed-rank tests, p < 0.05, Bonferroni corrected) between the first pulse and the participant-specific median of the later pulses (pulse numbers 2–30). For the active muscle, the level of the first pulse appears similar to the other pulses, and no significant difference could be demonstrated.

**Figure 4:**
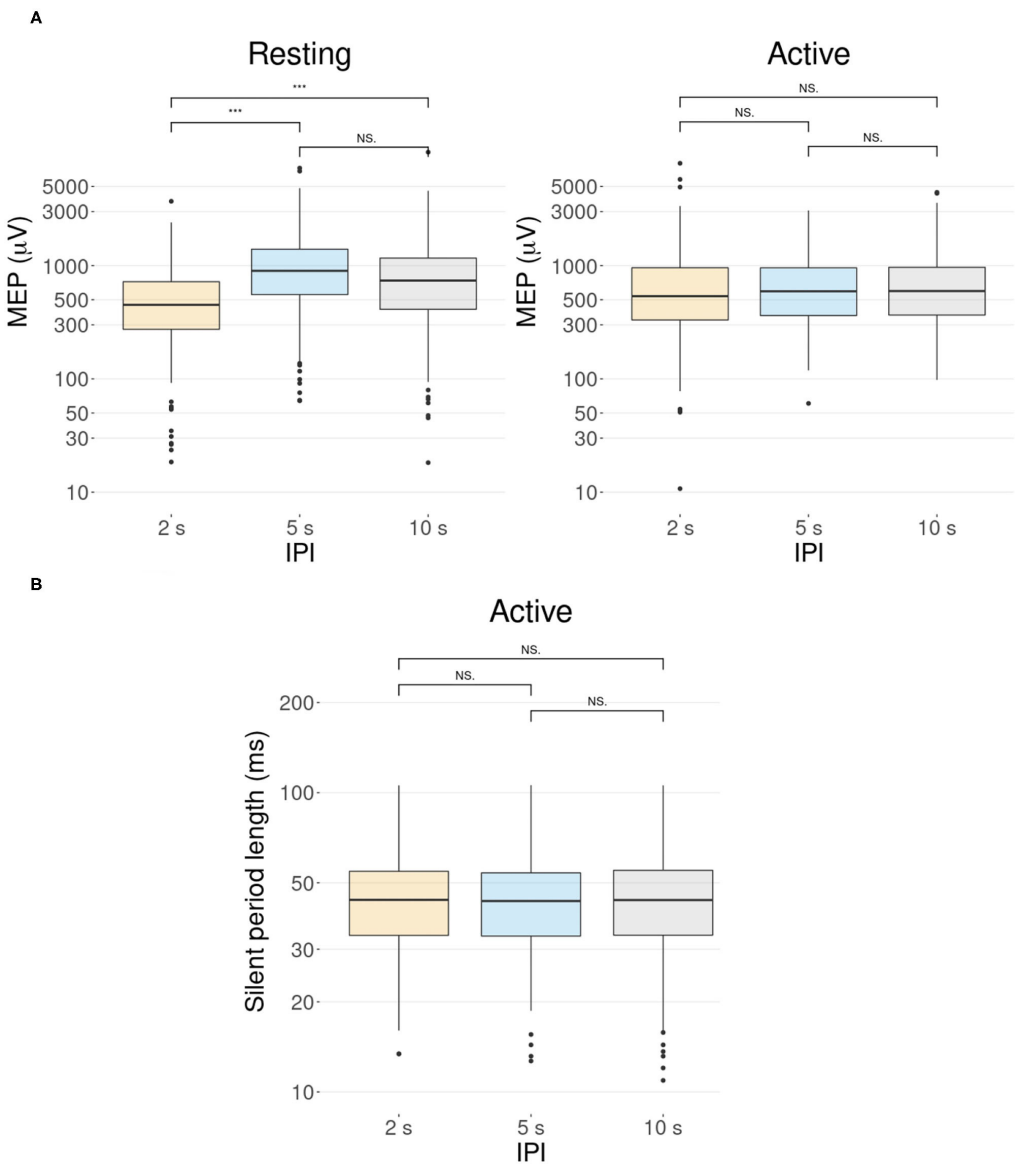
The effect of the IPI. Participant-specific intercepts have been removed from the data using a linear mixed effects model. Graphs represent the minimum, maximum, median, first quartile, and third quartile in the data set on a logarithmic scale. ***p ≤ 0.001, Ns, non-significance. **(A)** Pulses after the first pulse (pulse numbers 2–30) show a difference in the MEP amplitudes in the resting condition but not in the active condition. In the resting condition, the MEP amplitude of the 2 s IPI differs significantly from those of the 5 and 10 s IPIs (p < 0.001). In the active condition, there is no significant difference between the MEP amplitudes of different IPIs (p >0.1). **(B)** Boxplot illustration of the length of the silent period for different IPIs in the active condition. A Wilcoxon signed-rank test with a Bonferroni adjustment shows no support for significant differences between the different IPIs (all p = 1).

For later pulses (pulse numbers 2–30), there was a significant difference between the amplitudes of the 2 s IPI and the others (pairwise Wilcoxon signed-rank tests, p < 0.001, Bonferroni corrected) for the resting muscle (Figure 4A). On average, stimulation with the 2 s IPI decreased the median MEP amplitude by 14% compared to the 10 s IPI. For the active muscle, no significant differences (pairwise Wilcoxon signed-rank tests, all p >0.1) were found between the IPI groups of later pulses.

### 3.3. Effects of Inter-pulse Interval on Silent Period

A linear mixed-effects model with a likelihood ratio test for a silent period indicated that the IPI did not significantly affect the length of the silent period for the active muscle [χ^2^(2) = 0.11, p = 0.95]. Data is demonstrated in Figure 4B with *post-hoc* analysis showing no support for significant differences (pairwise Wilcoxon signed-rank tests, all p = 1, Bonferroni corrected) between the IPI groups.

## 4. Discussion

The current study investigated the effect of the TMS IPI on the MEP amplitude with the active and resting muscle. The objective was to find whether active muscle contraction could remove the modulatory effect of the IPI on the MEP amplitude, and therefore reduce the time used in TMS motor mapping.

Previous studies have found that, with a resting muscle, the MEP amplitudes were greatly dependent on the IPI (Möller et al., 2009; Julkunen et al., 2012; Vaseghi et al., 2015; Pellicciari et al., 2016; Hassanzahraee et al., 2019). The MEP amplitude was shown to increase as the IPI increased from 5 to 20 s (Möller et al., 2009), from 2 to 10 s (Julkunen et al., 2012), from 4 to 10 s (Vaseghi et al., 2015), and from 5 to 15 s (Hassanzahraee et al., 2019). Hassanzahraee et al. (2019) also showed that IPIs longer than 12 s did not differ in amplitude, as their IPI was sufficient for the recovery of the cerebral perfusion. Because of the recovery time, it is suggested to use a longer IPI when giving TMS with a resting muscle. Our findings support these previous results, as the shortest 2 s IPI significantly decreased the MEP amplitude compared to both 10 and 5 s IPIs. However, we could not show a significant difference between the 5 and 10 s IPIs, but the difference between them has been smaller than their difference with the 2 s IPI in previous studies as well. Furthermore, several rTMS studies (Chen et al., 1997; Siebner et al., 1999; Muellbacher et al., 2000) have reported that low-frequency rTMS on the motor cortex reduces cortical excitability with a resting muscle, which can be observed as a reduction of MEP amplitudes. This could also underlie the reduction of MEP amplitudes we observed with 2 s IPI, as the 2 s IPI is close to commonly used frequencies in low-frequency rTMS.

To our knowledge, there are no previous studies that have researched whether the IPI has an effect on the silent period. Our results indicate that there is no significant effect. Furthermore, we did not find significant effects of the IPI on the inter-trial variability of the MEP amplitude. However, the variability in the MEPs was significantly smaller with the active muscle compared to the resting muscle, which is due to the stabilization of corticospinal excitability through slight voluntary muscle contraction (Darling et al., 2006).

Our main finding indicates that MEPs with active muscle contraction during TMS are not affected by the IPI. A possible cause is that constant muscle contraction saturates the excitability of the corticomotorneurons, which prevents the recovery of the cerebral perfusion that is present with a resting muscle.

Our result allows the use of shorter IPIs in TMS studies in active muscles. This can significantly shorten the recording time, making measurement sessions more effective. Shortening the recording time is especially beneficial in studies where a large number of TMS pulses are used, such as novel computational–experimental techniques that have been developed for accurate localization of the activation sites of TMS (Bungert et al., 2017; Laakso et al., 2018; Weise et al., 2020; Kataja et al., 2021). These techniques rely on the computational analysis of the induced electric field and may require the application of more than a thousand TMS pulses. The use of a shorter IPI in active muscles could significantly improve the applicability of such methods.

The main drawback with all active muscle TMS studies is the inaccuracy of the constant muscle contraction force. This was also indicated by our results, which showed an effect of the baseline EMG signal magnitude on the MEP size, higher baselines producing larger MEPs. In order to secure a reliable muscle contraction of required level during stimulation, it is necessary to have a sufficient feedback method to ensure that the participant can maintain the correct contraction level. However, these methods are not feasible if the participant is not able for constant contraction of muscle or unable to contract the muscle at all. Additionally, active muscle contraction might be unsuitable for studies aiming to measure brain activity in combination with TMS, such as TMS-EEG studies (Ilmoniemi and Kičić, 2010), because the active contraction in itself affects the EEG. Moreover, we did not study the effect of IPIs shorter than 2 s, and therefore our result may not be applicable with shorter IPIs. Furthermore, the current study was mainly conducted in young adults and cannot be generalized to the entire population without critical judgement. It should also be noted that there where few participants who were different between the study conditions.

In conclusion, the present study revealed that active muscle contraction eliminates the modulating effect of the IPI that is present with a resting muscle. This result indicates that IPIs as short as 2 s can be used to speed up TMS motor mapping in active muscles. To our knowledge, this is the first study to compare the effects of three different IPIs on the MEP amplitude in both active and resting muscles.

## Data Availability Statement

The raw data supporting the conclusions of this article will be made available by the authors, without undue reservation.

## Ethics Statement

The studies involving human participants were reviewed and approved by Aalto University Research Ethics Committee. The patients/participants provided their written informed consent to participate in this study.

## Author Contributions

NM and IL designed the study. NM and MS performed the experiments. NM processed and analyzed the data and initially wrote the manuscript. IL obtained funding for the project. All authors reviewed, edited, and approved the final manuscript.

## Funding

This work was supported by the Academy of Finland (Grant Number 325326).

## Conflict of Interest

The authors declare that the research was conducted in the absence of any commercial or financial relationships that could be construed as a potential conflict of interest.

## Publisher's Note

All claims expressed in this article are solely those of the authors and do not necessarily represent those of their affiliated organizations, or those of the publisher, the editors and the reviewers. Any product that may be evaluated in this article, or claim that may be made by its manufacturer, is not guaranteed or endorsed by the publisher.
